# Refining anti‐inflammatory therapy strategies for bronchopulmonary dysplasia

**DOI:** 10.1111/jcmm.13044

**Published:** 2016-12-13

**Authors:** Ina Rudloff, Steven X. Cho, Christine B. Bui, Catriona McLean, Alex Veldman, Philip J. Berger, Marcel F. Nold, Claudia A. Nold‐Petry

**Affiliations:** ^1^ Ritchie CentreHudson Institute of Medical ResearchMelbourneVictoriaAustralia; ^2^ Department of PaediatricsMonash UniversityMelbourneVictoriaAustralia; ^3^ Department of Anatomical PathologyAlfred HospitalMelbourneVictoriaAustralia; ^4^ Central Clinical SchoolMonash UniversityMelbourneVictoriaAustralia

**Keywords:** IL‐1 receptor antagonist, protein C, inflammation, hyperoxia, neonatal lung disease

## Abstract

Bronchopulmonary dysplasia (BPD) is a severe lung disease of preterm infants, which is characterized by fewer, enlarged alveoli and increased inflammation. BPD has grave consequences for affected infants, but no effective and safe therapy exists. We previously showed that prophylactic treatment with interleukin‐1 receptor antagonist (IL‐1Ra) prevents murine BPD induced by perinatal inflammation and hyperoxia. Here, we used the same BPD model to assess whether an alternative anti‐inflammatory agent, protein C (PC), is as effective as IL‐1Ra against BPD. We also tested whether delayed administration or a higher dose of IL‐1Ra affects its ability to ameliorate BPD and investigated aspects of drug safety. Pups were reared in room air (21% O_2_) or hyperoxia (65% or 85% O_2_) and received daily injections with vehicle, 1200 IU/kg PC, 10 mg/kg IL‐1Ra (early or late onset) or 100 mg/kg IL‐1Ra. After 3 or 28 days, lung and brain histology were assessed and pulmonary cytokines were analysed using ELISA and cytokine arrays. We found that PC only moderately reduced the severe impact of BPD on lung structure (e.g. 18% increased alveolar number by PC *versus* 34% by IL‐1Ra); however, PC significantly reduced IL‐1β, IL‐1Ra, IL‐6 and macrophage inflammatory protein (MIP)‐2 by up to 89%. IL‐1Ra at 10 mg/kg prevented BPD more effectively than 100 mg/kg IL‐1Ra, but only if treatment commenced at day 1 of life. We conclude that prophylactic low‐dose IL‐1Ra and PC ameliorate BPD and have potential as the first remedy for one of the most devastating diseases preterm babies face.

## Introduction

First described in 1967 1, BPD is the most common chronic lung disease affecting preterm infants. BPD incidence exceeds 50% in the most premature infants before falling with increasing gestational age at birth 2. As modern medicine is increasingly adept at keeping extremely preterm babies alive 3, BPD is on the rise.

BPD used to be characterized by fibrosis resulting from ventilation‐induced injury of the delicate lung tissue. With the introduction of gentle ventilation methods, antenatal steroids and surfactant‐replacement therapy, the phenotype of BPD has changed dramatically. Nowadays, the ‘new BPD’ is characterized by arrested lung development involving reduced alveolarization and vascularization 4. Affected children suffer from impaired neurodevelopment 5, respiratory dysfunction 6 and susceptibility to airway infections 7 that can deteriorate suddenly and even lead to death. As BPD‐associated lung damage is irreversible, the complications of the disease can persist into adulthood 8, 9. Thus, BPD has a substantial impact on the quality of life of those directly affected and their families.

The ultimate mechanism of BPD development is pulmonary inflammation, which may be triggered by insults like mechanical ventilation, antenatal and post‐natal infections and hyperoxia 10, 11. Pro‐inflammatory cytokines, including interleukin (IL)‐1β, IL‐6 and tumour necrosis factor (TNF), are associated with BPD and adverse outcome such as prolonged oxygen requirement and hospitalization 12, 13, 14. Recognizing that BPD is an inflammatory disease, the American Academy of Pediatrics (AAP) called for escalated research into new anti‐inflammatory strategies 2, but no effective and safe therapy has yet emerged. Heeding the academy's call, we focused on inhibition of IL‐1 as a potential therapeutic approach. We showed that at a dose of 10 mg/kg, the endogenous inhibitor of IL‐1, interleukin‐1 receptor antagonist (IL‐1Ra), ameliorates murine BPD induced by perinatal inflammation and hyperoxia 15. To advance the prospects for an anti‐inflammatory treatment, we now extend our earlier study by examining whether the benefit of IL‐1Ra on BPD improves at a dose higher than 10 mg/kg. We also assess IL‐1Ra's effectiveness if treatment is postponed to post‐natal day 6 when symptoms of BPD are usually evident in premature infants.

In an attempt to add to the limited therapies available for treating BPD, we furthermore explore the effectiveness of a second anti‐inflammatory molecule, protein C (PC), a serine protease that upon cleavage is converted into its active form, aPC 16. The distinction between PC and aPC is important, as unlike recombinant PC, recombinant aPC proved to be ineffective in sepsis trials 17 and has been withdrawn from the market. Therefore, in this study, we investigated PC and not its activated form as a potential therapeutic for BPD. Traditionally considered an anticoagulant 18, the potent anti‐apoptotic, cyto‐protective and anti‐inflammatory actions of PC are well‐recognized 19, 20, 21. PC achieves its anti‐inflammatory action partly through inhibition of pro‐inflammatory cytokines such as IL‐1β and IL‐6, and by increasing the production of the anti‐inflammatory IL‐10 22, 23, 24, 25. As an established treatment of human diseases such as congenital protein C deficiency, PC has proven its safety in children; and of note, PC has also been successfully and safely used to treat neonatal sepsis 26, 27, 28, 29.

## Materials and methods

### Ethics

All experimental procedures conformed with the guidelines established by the National Health and Medical Research Council, had the approval of Monash University's Ethics Committee (MMCA/2010/05, MMCA/2011/35 and MMCA/2013/45) and complied with the Declaration of Helsinki.

### Murine model of BPD

**Figure 1:**
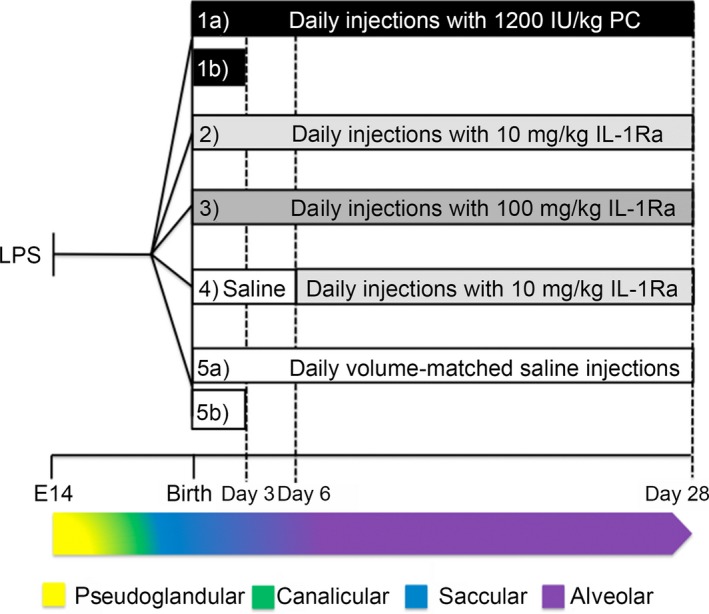
Overview of the BPD model and experimental set‐up. Pregnant dams were injected i.p. with lipopolysaccharide (LPS) at day 14 of gestation (embryonic day 14, E 14). Within 24 hrs after birth, pups were randomly allocated into five treatment groups and were either housed in hyperoxia (65% O_2_ or 85% O_2_) or room air (21% O_2_). The five treatment groups were 1) daily subcutaneous (s.c.) injections with 1200 IU/kg PC for 28 days (1a) or 3 days (1b); 2) daily s.c. injections with 10 mg/kg IL‐1Ra for 28 days; 3) daily s.c. injections with 100 mg/kg IL‐1Ra for 28 days; 4) daily s.c. injections with saline until day 6, then daily s.c. injections with 10 mg/kg IL‐1Ra until day 28 (referred to as late onset treatment); and 5) daily s.c. injections with volume‐matched saline for 28 days (5a) or 3 days (5b). Pups were humanely killed at day 3 for pulmonary cytokine analysis or at day 28 for analysis of lung and brain structure. As described in the study of Backstrom E *et al*. (2011), our experimental set‐up comprised four of the five stages of the murine lung development. The embryonic stage, not depicted here, occurs in the mouse between E 9 and 11.5 and is equivalent to 3–7 gestational weeks (GW) in humans. Depicted are the pseudoglandular (E 11.5–16.5 and GW 5–17), canalicular (E 16‐18 and GW 16–26), saccular (E 17.5‐ post‐natal day 5 and GW 24–38) and alveolar phase (post‐natal days 5–28 and >GW 36).

As previously reported 15, BPD was induced by a two‐pronged approach. We subjected pregnant C57BL/6J mice on day 14 of gestation to intraperitoneal (i.p.) injection with 150 μg/kg of lipopolysaccharide (LPS) to mimic maternal systemic inflammation (e.g. chorioamnionitis). Pups were born at term (days 19–21 of gestation) when their lungs are at the saccular stage 30, comparable to a premature human baby of 24–38 weeks’ gestational age 31. Within 24 hrs after birth, pups and dams were randomized to be exposed to gas with an FiO_2_ of 0.21 (room air), 0.65 (hyperoxia 65%) or 0.85 (hyperoxia 85%) for 3 or 28 days. Pups were also randomly allocated into treatment groups: (*i*) daily subcutaneous (s.c.) injections for 3 or 28 days of human PC (1200 IU/kg; this dose is 12 times higher than that used in humans [100 IU/kg, Ceprotin protein C concentrate prescribing information, Baxter] compensating for the species‐specific differences in activity as per US Food and Drug Administration (FDA) recommendations), (*ii*) IL‐1Ra 10 mg/kg or (*iii*) IL‐1Ra 100 mg/kg, s.c. injections for 28 days, (*iv*) 10 mg/kg IL‐1Ra from day 6 to day 28 (late onset) or (*v*) s.c. injections with an equal volume of saline (vehicle). Temperature (22°C) and humidity (50–60%) were kept constant, and light was cycled in a 12 hrs day/night rhythm. Dams had unrestricted access to food and water and were rotated between room air and hyperoxia groups in a 3 days cycle to protect them from prolonged hyperoxia. Dams remained healthy with no effects from exposure to hyperoxia. Bodyweights of pups reared in room air were normal (compared to litters from dams not exposed to hyperoxia) indicating that hyperoxia did not affect lactation in dams. At 3 or 28 days, pups were humanely killed and lungs and brains processed. For an overview of treatment conditions, see Figure 1.

### Activation of human PC in mice

Untreated 28‐day‐old mice were injected s.c. with human PC (1200 IU/kg) or vehicle (volume‐matched). 60 min. later, plasma aPC concentration was measured using a human aPC ELISA.

### Lung preparation and histology

After cervical dislocation, we intubated 28‐day‐old mice *via* the trachea, tied the left main bronchus and removed the left lung for molecular analysis. We fixed the right lung with 4% PFA (pH 7.4, instilled at 20 cmH_2_O pressure), dissected it free, placed it in 4% PFA for ≥2 hrs and processed it for paraffin embedding. Lung tissue was cut into 3 μm sections and subsequently H&E‐stained. The following groups comprise a combination of 28‐day lungs obtained specifically for this study and, for direct comparison, a re‐analysis of lungs included in Nold MF *et al*. 15: air vehicle, hyperoxia vehicle, air IL‐1Ra 10 mg/kg (early onset) and hyperoxia IL‐1Ra 10 mg/kg (early onset).

### Analysis of lung structure

Using an Aperio Scanscope (ePathology Solutions, Leica Biosystems, Nussloch, Germany), sections were scanned at 200‐fold magnification. Whole lung images were analysed (ImageJ (NIH)) to quantify alveolar number, size and surface area‐to‐volume ratio. A macro was generated for lung analysis in ImageJ, measuring and counting alveoli and their averages size in the range of 100–50,000 μm^2^. The lower gate excluded under‐inflated airspaces and blood vessels, while the upper gate excluded over‐inflated alveoli and larger airways. Additionally, we cross‐checked the outcome of the ImageJ analysis by comparing the computerized lung image with the original scan of the H&E slide to manually confirm the results.

### Brain preparation and histology

After removing the lungs from 28‐day‐old mice, we opened the skull and immediately placed the brain in 4% PFA for a minimum of 24 hrs before processing. The whole brain, minus the hindbrain, was embedded in paraffin. Sections (7 μm) were cut to expose the landmark of the hippocampal fissure (to interaural −0.08 mm and bregma −3.8) as described Paxinos G and Franklin KBJ 32. Sections were H&E‐stained and assessed by an animal pathologist.

For detection of cleaved caspase‐3 by immunohistochemistry, antigen retrieval was performed with Dako PT Link (Agilent Pathology Solutions Santa Clara, CA, USA) at 98°C in Dako Target Retrieval Solution for 30 min. The subsequent immunohistochemical staining was performed with a Dako Autostainer Plus: endogenous peroxidase activity was inhibited with Dako REAL Peroxidase‐Blocking Solution which was applied to the slides for 10 min. followed by a 10 min. blocking step performed with Dako Protein Block. Slides were then incubated with a rabbit anti‐mouse cleaved caspase‐3 antibody (1:200, cell signalling) for 90 min. at room temperature. Secondary antibody incubation and diaminobenzidine staining were performed with a Dako HRP‐labelled polyclonal anti‐rabbit antibody (60 min.) and Dako Liquid DAB+ Chromogen System (10 min.). Sections were counterstained with Dako REAL Hematoxylin and scanned on an Aperio Scanscope (ePathology Solutions).

### Tissue preparation for cytokine analysis

At day 3, lungs were removed, washed in ice‐cold sterile saline, snap‐frozen in liquid nitrogen and stored at −80°C. For cytokine analysis, lungs were homogenized in lysis buffer Petry C *et al*. 33 using an Ultra Turrax (IKA, Staufen, Germany). The lysate was centrifuged (10 min., 14,000 × g) and total protein concentration and cytokines assayed in the supernatants by ELISA and cytokine protein array. As the 3‐day PC experiments were performed simultaneously with the 3‐day IL‐1Ra experiments in Nold MF *et al*. 15, the air vehicle and hyperoxia vehicle control groups are identical to those shown in Figure 4 in Nold MF *et al*. 15.

### ELISA

Protein concentrations were quantified by ELISA according to the manufacturer's instructions (R&D Systems (Minneapolis, MN, USA), MIP‐2; BD (Franklin Lakes, NJ, USA), IL‐6; US Biological (Salem, MA, USA), aPC). Cytokine abundance was normalized to total protein concentration (determined by BCA assay; Thermo Fisher Scientific, Waltham, MA, USA).

### Cytokine protein array

Proteome Profiler Arrays (R&D Systems) were performed 34 by incubating equal volumes of tissue lysates with pre‐coated cytokine array membranes as specified by the manufacturer. Densitometry was performed and per cent changes in individual cytokines expressed as background‐corrected optical density (OD) per mm^2^. We controlled for loading variability between membranes by normalizing the density of each cytokine to the mean density of positive controls on the membranes.

### Statistical analysis

Groups were tested for normality and equal variance (p to reject 0.05) using GraphPad Prism 6.0e (GraphPad Software, San Diego, CA, USA). Thereafter, we performed Mann–Whitney *U*‐tests if only two groups were compared. We used Kruskal–Wallis or two‐way anova when multiple groups were compared. If a significant effect was revealed, Tukey's or Dunn's comparisons were performed (threshold for significance *P* < 0.05).

## Results

### IL‐1Ra‐dosage and timing are crucial

Having shown that IL‐1Ra prevents the development of murine BPD 15, we set out to probe dosage and timing effects of IL‐1Ra on BPD induced by perinatal inflammation and hyperoxia. Figure 1 provides an overview of the experimental design, treatment groups and relationship between the experimental time‐points and murine lung development.

**Figure 2:**
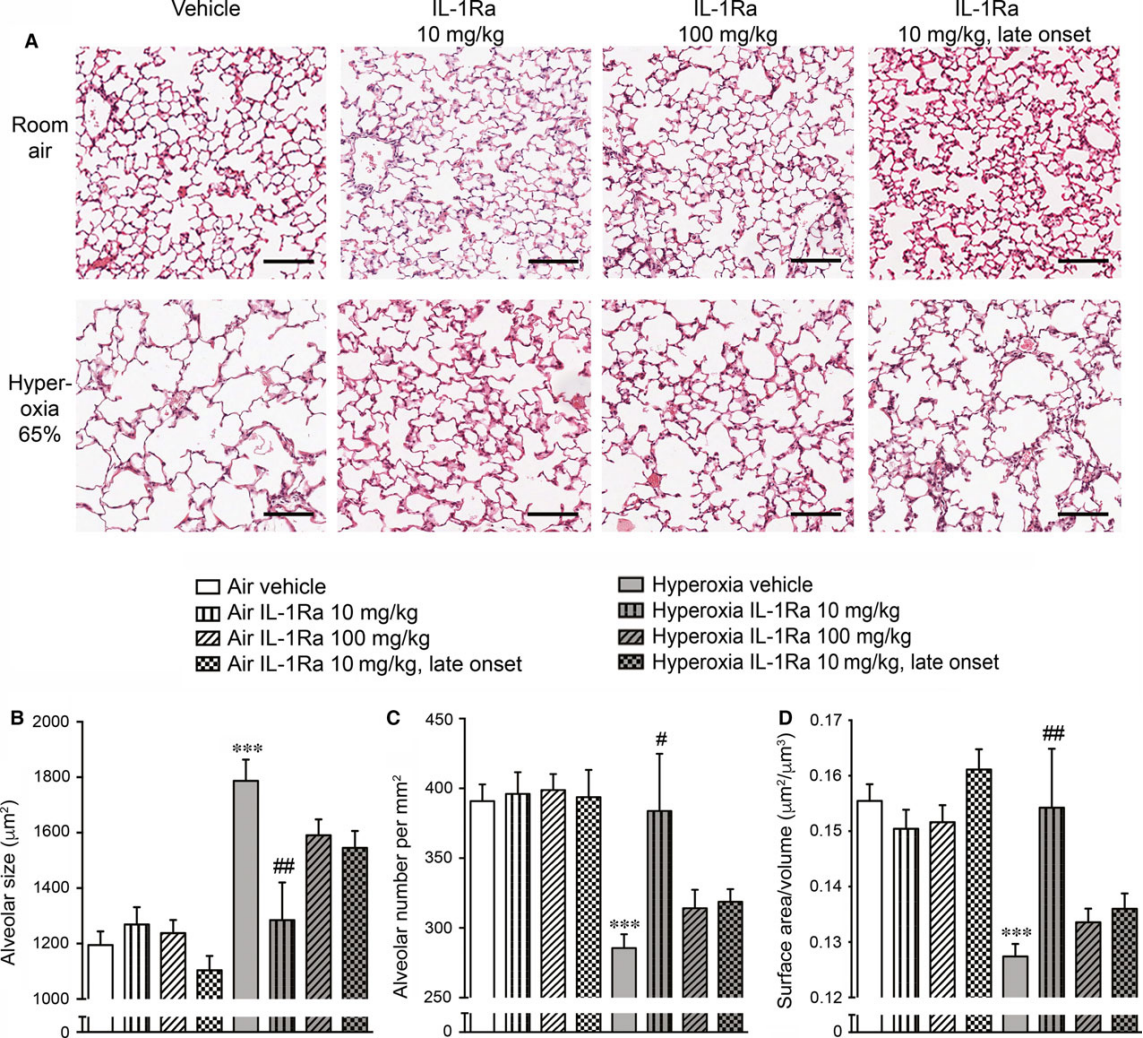
Lung histology and analysis of lung morphology in 28‐day‐old pups after perinatal LPS and post‐natal hyperoxia at 65%. At day 14 of gestation, pregnant dams were injected intraperitoneally with 150 μg/kg LPS. After delivery, newborn pups were exposed to 21% O_2_ (room air) or 65% O_2_ (hyperoxia) and received daily s.c. injections of either vehicle, 10 mg/kg IL‐1Ra or 100 mg/kg IL‐1Ra from day 1 to day 28. Another group received daily s.c. injections of vehicle from day 1 to day 5 followed by s.c. injections of 10 mg/kg IL‐1Ra from day 6 to day 28 (late onset). For all pups, lung morphology was assessed at day 28, n = 5–27 per group. (**A**) One representative image per group is shown. Scale bars 100 μm, ×200 magnification. (**B‐D**) Scans of whole lungs were analysed for alveolar size in μm^2^ (**B**), number of alveoli per mm^2^ (**C**) and the surface area‐to‐volume ratio (μm^2^/μm^3^) (**D**). Data are shown as mean ± S.E.M. ***P < 0.001 for air vehicle *versus* hyperoxia vehicle; #*P* < 0.05 and ##*P* < 0.01 for hyperoxia vehicle *versus* hyperoxia 10 mg/kg IL‐1Ra. LPS: lipopolysaccharide, IL‐1Ra: interleukin‐1 receptor agonist.

As shown in Figure 2A, mice exposed to antenatal inflammation and hyperoxia at 65% O_2_ for 28 days displayed BPD‐like changes in lung morphology as characterized by enlarged alveoli and sparse secondary septation. As we reported previously 15, daily injections with 10 mg/kg IL‐1Ra for 28 days almost completely prevented these morphological changes. To investigate whether increasing the IL‐1Ra dosage has further benefit, we injected newborn pups daily with 100 mg/kg IL‐1Ra until day 28 of life. Additionally, we assessed the effects of delayed IL‐1Ra treatment onset: pups received daily injections of vehicle until day 5, followed by daily IL‐1Ra injections (10 mg/kg) from day 6 to day 28. However, treatment with 100 mg/kg IL‐1Ra or delayed treatment with 10 mg/kg IL‐1Ra visually appeared not to be as effective in preventing BPD as prophylactic treatment with 10 mg/kg IL‐1Ra from day 1 to day 28. Indeed, quantitative analysis (Fig. 2B–D) revealed that compared to vehicle‐treated mice exposed to antenatal inflammation and housed in room air (21% O_2_), lungs from vehicle‐treated animals in hyperoxia exhibited a 50% increase in alveolar size (Fig. 2B), a 27% reduction in alveolar number per mm^2^ (Fig. 2C) and a 18% decrease in the surface area‐to‐volume ratio (Fig. 2D). These changes were almost completely prevented by prophylactic treatment with 10 mg/kg IL‐1Ra for 28 days (28% decrease in alveolar size, 34% increase in alveolar number and 21% increase in surface area‐to‐volume ratio compared to vehicle‐treated mice in hyperoxia). However, by day 28, neither high‐dose IL‐1Ra injections nor lateonset treatment was superior to daily treatment with 10 mg/kg IL‐1Ra from day 1 to day 28 after birth. In fact, both modifications of our standard protocol did not improve alveolar size (Fig. 2B), alveolar numbers (Fig. 2C) and surface area‐to‐volume ratio (Fig. 2D) compared to animals continuously receiving 10 mg/kg IL‐1Ra until day 28. These data demonstrate that a low dose (10 mg/kg), but not a 10‐fold higher dose (100 mg/kg), of IL‐1Ra is effective in protecting mouse pups from BPD and that to gain benefit, an early onset of treatment is imperative.

### PC ameliorates murine BPD induced by 65% O_2_

**Figure 3:**
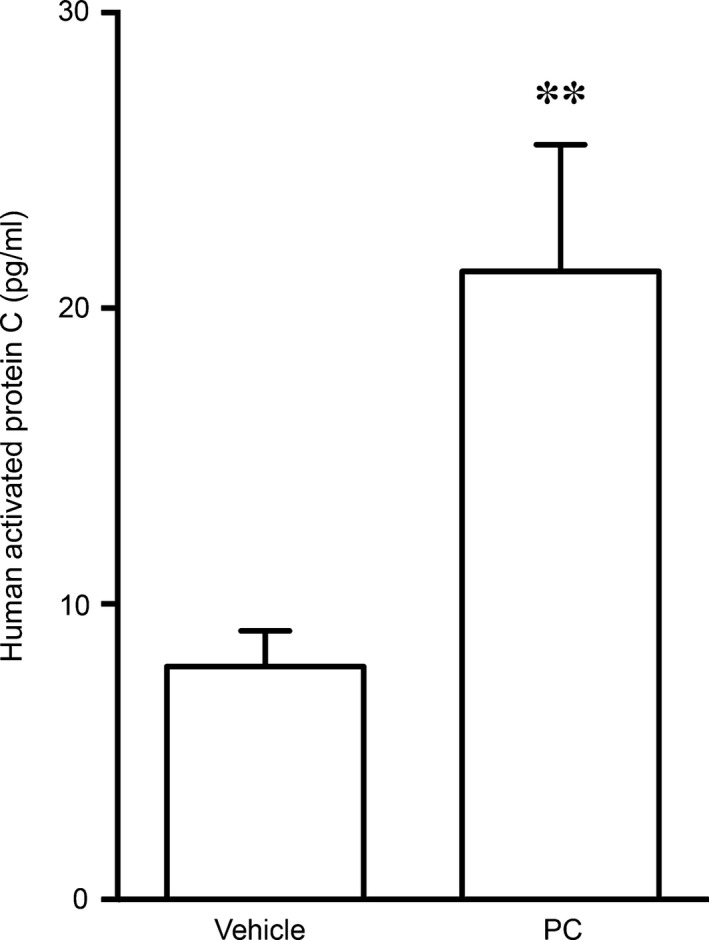
Activation of human PC in mice. Healthy 28‐day‐old mice were injected s.c. with 1200 IU/kg PC or vehicle (volume‐matched). 60 min. after injection, human aPC was measured in the plasma, n = 9–14 per group. Data are shown as means ± S.E.M. **P < 0.01 for vehicle *versus* PC. PC: protein C, aPC: activated protein C.

In the first experiments on PC, we examined whether mice convert human PC into its active form, aPC. In 28‐day‐old healthy mice injected with 1200 IU/kg PC, we detected 21 pg/ml of human aPC in the plasma 60 min. after injection (Fig. 3). Human aPC was also detectable in vehicle‐injected animals. However, this background human aPC abundance is likely due to interspecies cross‐reactivity of the human aPC ELISA.

**Figure 4:**
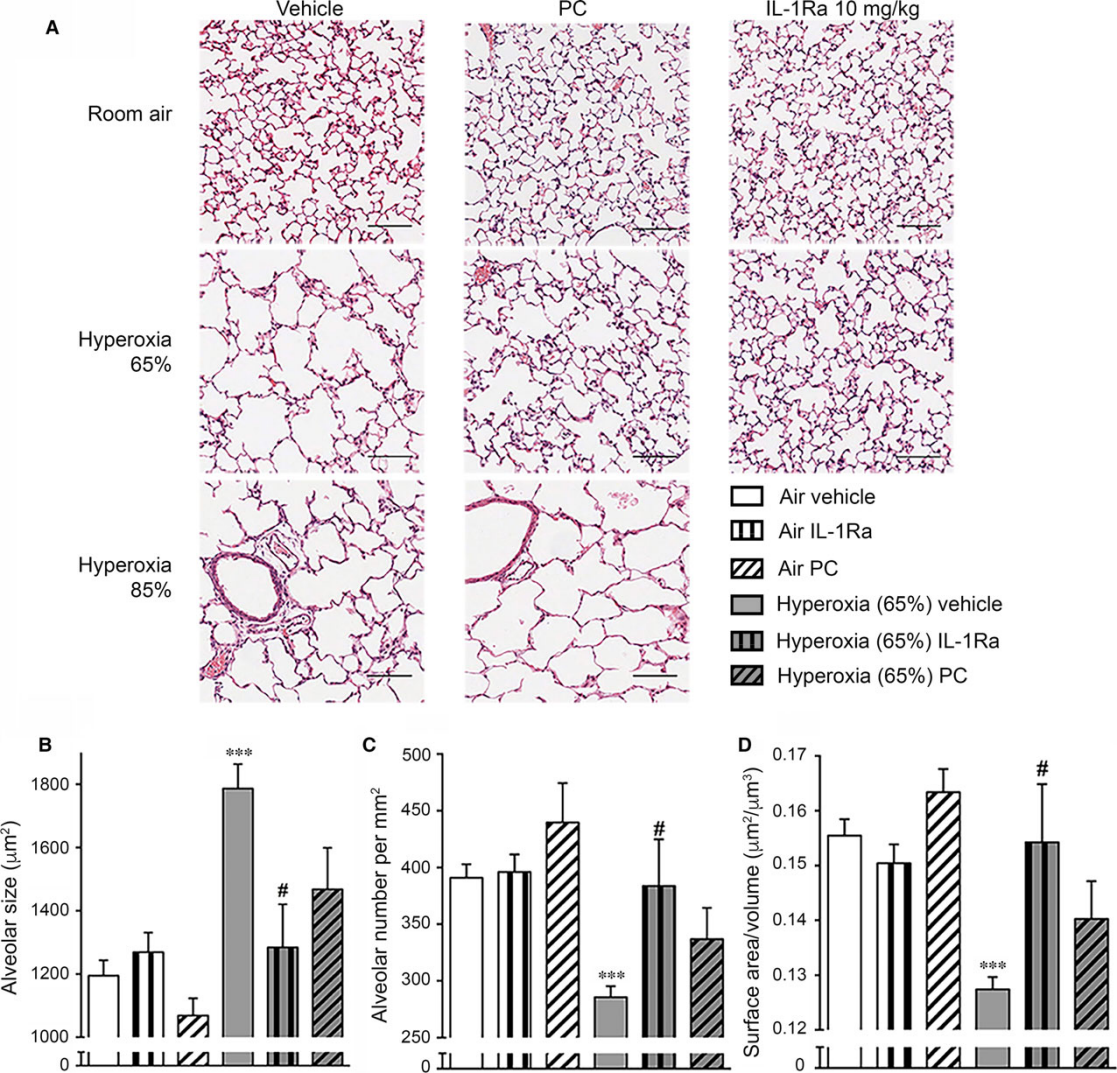
Lung histology at day 28 of life after antenatal LPS and exposure to hyperoxia. Pregnant dams were injected with 150 μg/kg LPS at day 14 of gestation. Newborn pups were subjected to 21% O_2_ (room air), 65% O_2_ or 85% O_2_ and received daily s.c. injections of vehicle, IL‐1Ra (10 mg/kg, early onset) or PC (1200 IU/kg). Vehicle‐ and IL‐1Ra‐injected animals in air and 65% O_2_ are identical with those shown in Fig. 2 as experiments depicted in Fig. 2 and Fig. 4 were carried out in parallel. The effects of IL‐1Ra treatment on animals reared in 85% hyperoxia are published in the study of Nold MF *et al*. 15 and thus are not depicted here. Lungs were analysed on day 28, n = 4–27 per group. (**A**) One representative slide per group is depicted. Scale bars 100 μm, ×200 magnification. (**B‐D**) Quantitative analysis of lung histology at 65% O_2_. Depicted are alveolar size in μm^2^ (**B**), number of alveoli per mm^2^ (**C**) and the surface area‐to‐volume ratio (μm^2^/μm^3^) (**D**). Data are represented as means ± S.E.M. ***P < 0.001 for air vehicle *versus* hyperoxia 65% vehicle; #*P* < 0.05 for hyperoxia 65% vehicle *versus* hyperoxia 65% IL‐1Ra. LPS: lipopolysaccharide, IL‐1Ra: interleukin‐1 receptor antagonist, PC: protein C.

Next, we investigated the effect of daily PC or vehicle injections on BPD development in pups exposed to antenatal inflammation and hyperoxia. At day 28, vehicle‐injected animals reared in hyperoxia (65% or 85% O_2_) exhibited a BPD‐like lung architecture with enlarged, simplified alveoli and meagre secondary septation (Fig. 4A). Prophylactic PC treatment mildly reduced the lung damage observed after 65% O_2_ exposure. In contrast, the BPD‐like phenotype induced by 85% O_2_ was not amenable to PC treatment. These data show that mice can convert human PC into aPC which then moderately improves BPD induced by a double hit of inflammation and 65% O_2_.

### PC is less effective in preventing murine BPD than IL‐1Ra

Next, we compared the effects of PC to those mediated by early‐onset, low‐dose IL‐1Ra in BPD induced by perinatal inflammation and 65% O_2_. Notably, the efficacy of IL‐1Ra in mice reared at 85% O_2_ was not tested, as we have previously shown that 10 mg/kg of IL‐1Ra did not ameliorate BPD at 85% O_2_ 15. Compared to vehicle‐treated pups in hyperoxia, prophylactic treatment with both IL‐1Ra and PC restored what visually appeared to be a near‐normal lung structure (Fig. 4A). Quantitative analysis revealed that there was a trend towards structural improvement upon treatment of mice with PC (Fig. 4B–D), but this effect was not statistically significant. In contrast to IL‐1Ra, PC did not significantly improve alveolar size (decrease of 18% with PC *versus* 28% with IL‐1Ra, Fig. 4B), alveolar number (increase of 18% with PC *versus* 34% with IL‐1Ra, Fig. 4C) and surface area‐to‐volume ratio (elevation by 10% with PC *versus* 21% with IL‐1Ra, Fig. 4D) compared to vehicle‐treated hyperoxia animals. Thus, prophylactic treatment with PC is not as effective as treatment with IL‐1Ra at the concentrations tested in this study.

### PC inhibits a set of pro‐inflammatory mediators

**Figure 5:**
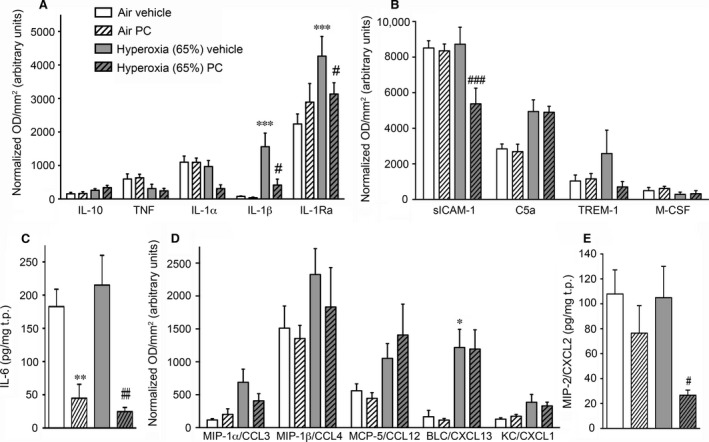
Immune profiling on day 3 of the murine BPD model with hyperoxia at 65% O_2_. Following the same experimental protocol as in Fig. 4, cytokines were detected in the lungs at day 3. Experiments were performed in parallel with those published in Fig. 4 in Nold MF *et al*. 15; thus, control animals (air vehicle and hyperoxia vehicle animals) are identical to those in Nold MF *et al*. 15. (**A,B,D**) Semi‐quantitative protein analysis of cytokines (**A**), other mediators (**B**) and chemokines (**D**) was performed by cytokine protein array, n = 3 per group. Data are shown as optical density (OD) normalized to the positive control spots on each membrane in arbitrary units ±S.E.M. (c,e) IL‐6 (c) and MIP‐2 (**E**) were measured by ELISA, n = 8–20 per group. Graphs show means of cytokine abundance normalized to total protein (t.p.) ±S.E.M. (**A‐E**) *P < 0.05, **P < 0.01 and ***P < 0.001 compared to air vehicle; #*P* < 0.05, ##*P* < 0.01 and ###*P* < 0.001 compared to hyperoxia vehicle. MIP‐2: macrophage inflammatory protein.

As inflammation is a hallmark of BPD, we investigated the effects of PC on pulmonary inflammatory markers at day 3 by assessing cytokines (Fig. 5A and C), chemokines (Fig. 5D and E) and other mediators of inflammation (Fig. 5B). Of note, the experiments shown in Figure 5 were carried out in parallel with those already published 15; thus, the same control animals (air vehicle and hyperoxia vehicle animals) were used in both studies. Compared to the pups in room air, subjecting animals to hyperoxia significantly increased concentrations of the mediators IL‐1β, IL‐1Ra and B lymphocyte chemoattractant (BLC)/CXCL13 (note that antenatal LPS caused a marked increase in most of the mediators tested here, see 15). Unexpectedly, PC treatment had little effect on the majority of pulmonary immune mediators investigated. However, PC‐injected pups in hyperoxia showed a significant drop in IL‐1β (by 73%), IL‐1Ra (27%), sICAM‐1 (38%), IL‐6 (89%) and MIP‐2/CXCL2 (75%) and exhibited a trend towards a decrease in IL‐1α, TREM‐1 and MIP‐1α. These results show that the pulmonary inflammation of murine BPD is amenable only in part to PC treatment.

### Post‐natal administration of PC (1200 IU/kg) and IL‐1Ra (10 mg/kg or 100 mg/kg) does not affect brain development of newborn mice

**Figure 6:**
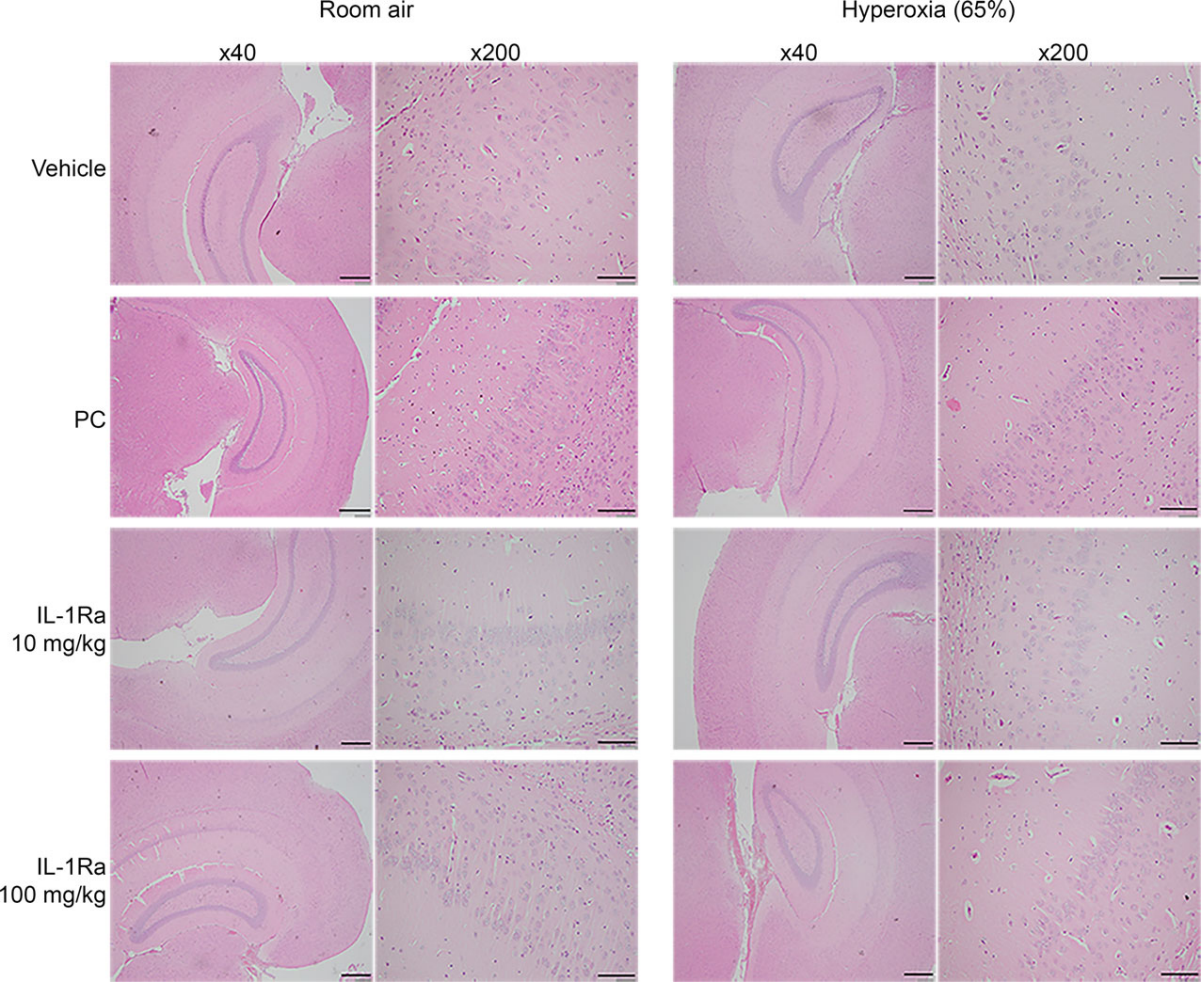
Hippocampus morphology at day 28. Pregnant dams were injected with 150 μg/kg LPS at day 14 of gestation. Newborn pups were reared in 21% O_2_ (room air) or 65% O_2_ (hyperoxia) and received daily s.c. injections of vehicle, PC (1200 IU/kg) or IL‐1Ra (10 mg/kg or 100 mg/kg). Brains were H&E‐stained and analysed on day 28, n = 3–24 per group. One representative slide per treatment group showing the pyramidal layer is depicted at low (×40) and high (×200) magnification. Scale bars: 400 μm for ×40 magnification, 100 μm for ×200 magnification. LPS: lipopolysaccharide, PC: protein C, IL‐1Ra: interleukin‐1 receptor antagonist.

We investigated whether cerebral morphological abnormalities such as apoptosis, loss of neurons, gliosis, white matter changes or inflammation occur during treatment with PC or IL‐1Ra. An experienced neuropathologist (blinded to the treatment) analysed H&E‐stained sections of the cerebrum, cerebellum and brain stem (Fig. 6), as well as similar sections stained for cleaved caspase‐3 as marker for apoptosis 35 (Fig. S1). Apoptosis was not detectable in any of the brain sections assessed. Moreover, neither the cortical neuronal lamination, nor temporal cortical width at the level of the hippocampus, nor white matter myelination extent and maturation showed any significant variation between treatment groups. There was no indication for haemorrhage, loss of neurons, gliosis, inflammation or other significant morphologic abnormalities.

## Discussion

BPD is a serious inflammation‐driven disease of preterm infants with currently no accepted safe and effective treatment. Our study aimed to advance the prospects for anti‐inflammatory agents to prevent or ameliorate BPD. We expanded on our earlier finding that IL‐1Ra is an effective treatment for BPD by examining the impact of a higher dose and delayed treatment onset. We also investigated whether an alternative anti‐inflammatory agent, PC, has beneficial effects on BPD development.

We already showed the promise of IL‐1Ra to prevent murine BPD when injected daily at 10 mg/kg starting from the day of birth 15. In the current study, we addressed three issues: first, whether the clinical efficacy of IL‐1Ra is reduced when treatment is delayed; second, whether the observed IL‐1Ra‐mediated inhibition of murine BPD is greater at a higher dose; and third, whether post‐natal treatment with IL‐1Ra has adverse effects on brain morphology.

Mouse pups subjected to antenatal LPS and perinatal inflammation have massively increased pulmonary cytokines at day 3 of life 15, and it is known that infants with high concentrations of pro‐inflammatory cytokines in their bronchoalveolar lavage have a substantially elevated risk of developing chronic lung disease and BPD 13, 36, 37, 38. To investigate the impact of treatment delay, we started IL‐1Ra application at day 6 after birth, well after the onset of the perinatal cytokine storm, and at the time at which the pulmonary disease of prematurity is starting to reveal its course in human preterm infants. Notably, we found that delayed treatment with IL‐1Ra is not as effective in protecting from murine BPD as treatment initiated on day 1 of life. These findings confirm an earlier demonstration that immediate intervention is necessary to prevent excessive inflammatory responses to perinatal and early post‐natal inflammatory stimuli 2.

Assessing whether increasing the dose of IL‐1Ra further improves the lung abnormalities of BPD, we used 100 mg/kg, a concentration 10 times higher than our standard dose and 20 times higher than that approved by the FDA for infants with neonatal onset of multi‐inflammatory disease (NOMID) 39. Our data show that 100 mg/kg IL‐1Ra bestows no benefit above that obtained with 10 mg/kg in our BPD model. In neonatal rats, an even higher IL‐1Ra dose (500 mg/kg, a human equivalent of 80 mg/kg) provoked two undesirable effects. First, there was a large influx of macrophages into the lung 40, an effect not observed in our newborn mice treated with 10 mg/kg IL‐1Ra 15. Second, 500 mg/kg IL‐1Ra reduced the total number of lung vessels in newborn rats reared in air 40. Considering the data available, we conclude that 10 mg/kg IL‐1Ra, a dose 50‐fold lower than that reported to cause adverse effects in rat lungs, represents a maximally effective dose when treatment starts immediately after birth.

The third issue we addressed here is the report that interference with the IL‐1 signalling pathway (using knockout or transgenic animals) adversely affects brain development and function, including memory 41, 42. The view has developed that both too little IL‐1, and too much, have detrimental effects on the brain, leading to the concept that a balance of IL‐1/IL‐1Ra is crucial for normal development 43. When IL‐1 signalling has been absent from conception in homozygous transgenic mice overexpressing soluble IL‐1Ra, hippocampal volume is reported to be reduced 42, although correlative histological studies are not available. Obviously, if interference with the IL‐1/IL‐1Ra balance has a major impact on brain structure and function, IL‐1Ra would be unattractive as a therapy in early development. We consider there are strong reasons to doubt that temporary IL‐1Ra treatment poses a problem for the newborn. First, if loss of IL‐1 signalling is intermittent, as it would be when a blocking agent is used as a therapy, as opposed to continuous as in genetically modified animals, no adverse impact is seen on behaviour 42. Second, in mice treated daily with IL‐1Ra, we found no evidence of significant apoptosis or morphological changes in the brain at 28 days, neither in mice exposed to 10 mg/kg nor to 100 mg/kg IL‐1Ra. Our finding may result from the short IL‐1Ra half‐life in rodents 44, 45 and the restoration of physiological IL‐1Ra plasma concentrations within 24 hrs.

The likelihood that IL‐1Ra poses safety concerns in human infants also gains no support from case reports, which do not mention abnormalities in babies delivered from mothers treated with IL‐1Ra during pregnancy 46, 47. Moreover, there is no evidence of memory or cognitive decline in adult humans exposed to lengthy periods (>10 years) of treatment with IL‐1Ra 48, 49. Furthermore, it has been shown that twice‐daily subcutaneous injection of IL‐1Ra is neuroprotective in a perinatal model of foetal infection and post‐natal hypoxic ischaemia 50. Additionally, anakinra, which is the recombinant IL‐1Ra used for treating diseases such as rheumatoid arthritis, neonatal‐onset multi‐inflammatory disease and auto‐inflammatory diseases, has exhibited an excellent safety profile and high efficacy 49.

The obvious implication of our results is that treatment with IL‐1Ra must commence before the occurrence of the extensive inflammatory response that ultimately triggers BPD, and well before the disease is diagnosed. Thus, babies at high risk of developing BPD would have to receive IL‐1Ra as a preventative therapy. Importantly, unlike corticosteroids (currently the only drug available for BPD), IL‐1Ra has no adverse effect on the growth of the lung or other organs 51 in fact, in children with systemic juvenile idiopathic arthritis, IL‐1Ra application had the desirable outcome that steroid dosage could be reduced, resulting in an *accelerated* growth rate 48. Even though there is a considerable cost to implementing preventative treatment of preterm neonates with IL‐1Ra, sparing them from BPD would make a very substantial saving to health care and this is not taking substantial psychosocial benefits for the patients and their families into account. In view of its effectiveness, and a lack of serious side‐effects even at concentrations 10 times higher than our therapeutic dose, IL‐1Ra has promise as a prophylactic therapy for BPD. We acknowledge, however, that while addressing several major aspects of human BPD, our experiments left other areas such as vascular structure, extracellular matrix remodelling and other mediators such as growth factors unexplored. To further assess the potential of IL‐1Ra in the prevention of BPD, these aspects are subject to current and future work at our laboratory.

To broaden the anti‐inflammatory arsenal for treating BPD, we also evaluated the effectiveness of PC. On the basis of its anti‐inflammatory, anti‐apoptotic and membrane‐stabilizing properties, PC has been extensively studied *in vivo* and *in vitro* with a view to novel therapeutic applications 19, 24, 27, 52. In contrast to recombinant aPC, a potent systemic anticoagulant, the non‐activated PC preparation does not increase the propensity to bleed and is thus safe to use in children. Human PC concentrate has been licensed in children for congenital PC deficiency and has been used in term and preterm neonates and in children where it has a reassuring safety profile 26, 27, 28. Of note, we found no evidence of adverse effects of PC treatment on brain morphology. PC did not prevent the severe lung damage induced by perinatal inflammation and 85% hyperoxia, just as we showed for IL‐1Ra previously 15. We found that PC ameliorated the lung structural damage in murine BPD induced by 65% hyperoxia, but less effectively and significantly than IL‐1Ra. PC treatment did not have as broad an effect on pulmonary cytokine production as IL‐1Ra. IL‐1Ra mainly exerted its effects on the pro‐inflammatory IL‐1α, IL‐1β, TREM‐1, IL‐6, MIP‐1α and MIP‐2 (as discussed in the study of Nold MF *et al*. 15), whereas PC significantly reduced pulmonary IL‐1β, IL‐6, sICAM‐1 and MIP‐2. Importantly, PC did not significantly reduce lung TREM‐1 and MIP‐1α, which we earlier identified as key players in the development of murine BPD 15. Thus, it appears likely that PC and IL‐1Ra ameliorate murine BPD *via* different mechanisms. It is well known that IL‐1Ra directly inhibits IL‐1 signalling and thus the activation of JNK, p38 mitogen‐activated protein (MAP) kinase and the nuclear factor (NF)‐κB signalling pathway 53. The mechanisms underlying the anti‐inflammatory and protective functions of PC, however, are much broader. While activated PC inhibits p38 MAP kinase and NF‐κB 24, 25, it can also block the production of pro‐inflammatory cytokines by inhibiting the Wnt5A ‐ Ca^2+^/calmodulin‐dependent protein kinase (CaMKII) pathway 54. Moreover, anti‐apoptotic and protective functions of PC can be mediated by the activation of protease‐activated receptor 1 (PAR‐1) which in turn triggers downstream signalling pathways 55. The fact that PC and IL‐1Ra have different mechanisms of action might explain why PC was not as potent as IL‐1Ra in preventing the inflammation‐ and hyperoxia‐induced cytokine response on day 3 and subsequently the development of BPD in our model.

A key implication of our study is that to achieve maximum benefit with anti‐inflammatory agents, administration should commence as early as possible, that is before the first signs of evolving BPD arise. Thus, these data suggest that prophylactic treatment of babies at highest risk may be the most promising approach. Evidence that IL‐1Ra and PC exert their effects on distinct sets of immune mediators suggests that a combination therapy might be beneficial in preventing BPD; we will test this hypothesis in future studies. Taken together, given their excellent safety profile in adults, children and neonates, IL‐1Ra or a combination therapy with PC could brighten the future for the tiniest and most vulnerable of patients, preterm babies.

## Conflict of interest

This study was partly sponsored by Baxter Bioscience (funds to AV and PJB; BT09‐000092).

## Authorship statement

All authors were involved in drafting the article or revising it critically for important intellectual content, and all authors approved the final version to be published. Dr. Nold‐Petry had full access to all of the data in the study and takes responsibility for the integrity of the data and the accuracy of the data analysis. Veldman, Berger, Nold and Nold‐Petry were involved in study conception and design. Rudloff, Cho, Bui and Nold‐Petry participated in acquisition of data. Rudloff, Cho, Bui, McLean, Veldman, Berger, Nold and Nold‐Petry carried out the analysis and interpretation of data.

## Supporting information


**Figure S1** Staining for cleaved caspase‐3 in brains at day 28. At day 14 of gestation, pregnant dams were injected with LPS (150 μg/kg). Within 24 hrs after birth, pups were either allocated to room air (21% O_2_) or hyperoxia (65% O_2_) and injected daily (s.c.) with volume‐matched vehicle, PC (1200 IU/kg), or IL‐1Ra (10 mg/kg or 100 mg/kg). At day 28, brains were stained for cleaved caspase‐3 by immunohistochemistry and analysed (n = 3–5 per group). One representative slide per treatment group is depicted. Scale bars 100 μm, ×200 magnification. LPS, lipopolysaccharide; PC, protein C; IL‐1Ra, interleukin‐1 receptor antagonist.
