# Supplementary figures and images for: Refining anti‐inflammatory therapy strategies for bronchopulmonary dysplasia
Source: J Cell Mol Med. 2016 Dec 13;21(6):1128–38. doi: 10.1111/jcmm.13044 (PMC5431131; doi:10.1111/jcmm.13044)

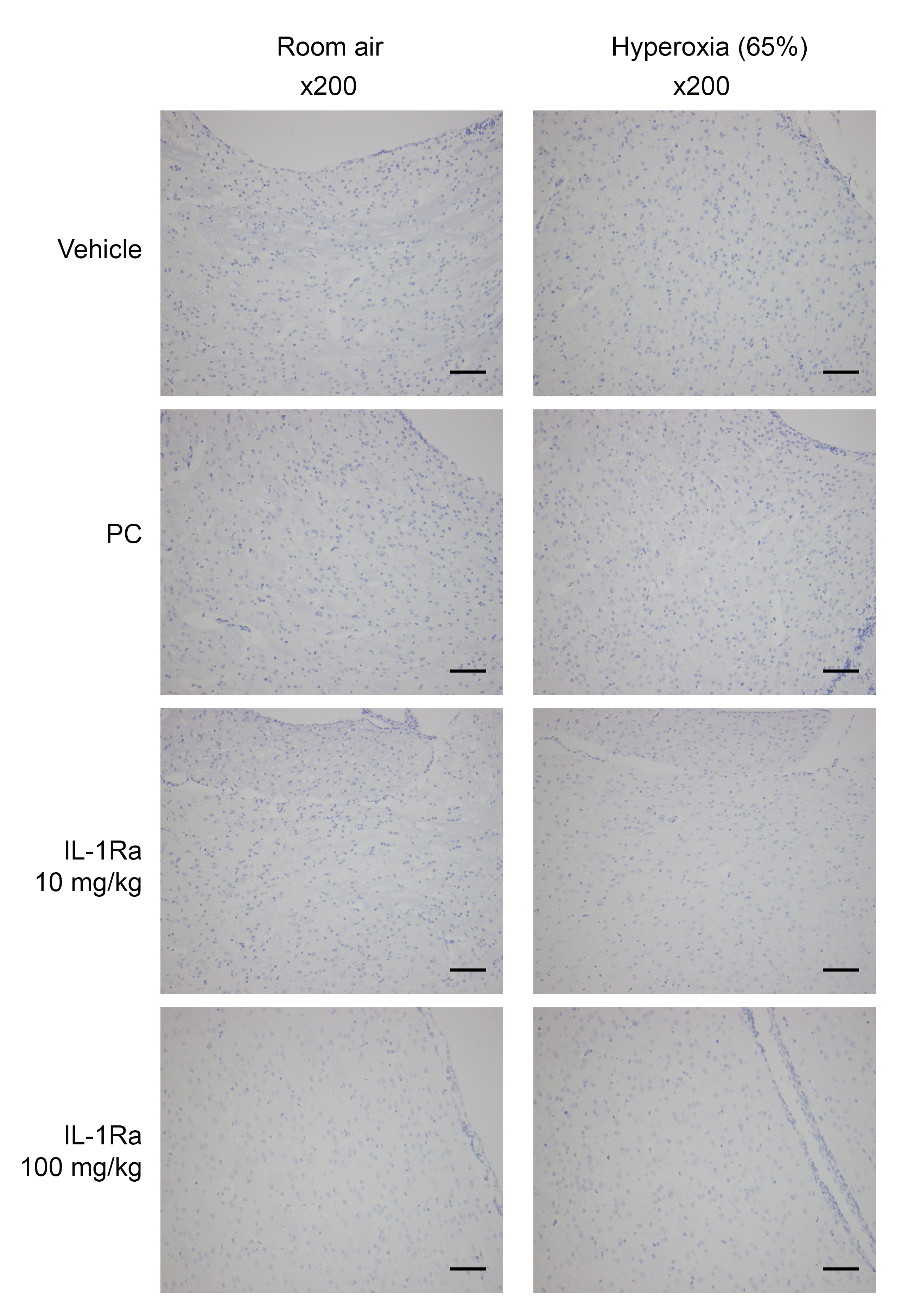

Supplement: Supplementary file 1 — Figure S1 Staining for cleaved caspase‐3 in brains at day 28. At day 14 of gestation, pregnant dams were injected with LPS (150 μg/kg). Within 24 hrs after birth, pups were either allocated to room air (21% O2) or hyperoxia (65% O2) and injected daily (s.c.) with volume‐matched vehicle, PC (1200 IU/kg), or IL‐1Ra (10 mg/kg or 100 mg/kg). At day 28, brains were stained for cleaved caspase‐3 by immunohistochemistry and analysed (n = 3–5 per group). One representative slide per treatment group is depicted. Scale bars 100 μm, ×200 magnification. LPS, lipopolysaccharide; PC, protein C; IL‐1Ra, interleukin‐1 receptor antagonist. [file JCMM-21-1128-s001.tif]
